# 240. Epidural Involvement Complicating Pyogenic Spondylodiscitis

**DOI:** 10.1093/ofid/ofab466.442

**Published:** 2021-12-04

**Authors:** Fatma Hammami, Makram Koubaa, Amal Chakroun, Khaoula Rekik, Chakib Marrakchi, Fatma Smaoui, Mounir Ben Jemaa

**Affiliations:** Infectious Diseases Department, Hedi Chaker University Hospital, University of Sfax, Tunisia, Sfax, Sfax, Tunisia

## Abstract

**Background:**

Pyogenic spondylodiscitis is an infection of the intervertebral disc(s) and/or adjacent vertebrae. It might be associated with epidural involvement. We aimed to study clinical, laboratory and evolutionary features of epidural involvement complicating pyogenic spondylodiscitis.

**Methods:**

We conducted a retrospective study including patients hospitalized for spondylodiscitis with epidural involvement in the infectious diseases department between 2007 and 2019.

**Results:**

We included 22 patients among whom 16 were males (72.7%). The mean age was 64±11 years. Eleven patients had diabetes mellitus (50%). The onset of the disease was acute in 18 cases (81.8%) and sub-acute in 4 cases (18.2%). The median delay to diagnosis was 4 [2-13] weeks. The revealing symptoms were back pain (95.5%), fever (68.2%) and asthenia (54.5%). Motor deficit was noted in 9 cases (40.9%), sensory deficit in 4 cases (18.2%) and sphincter dysfunction in one case (4.5%). Physical examination revealed spinal tenderness (77.3%), paravertebral tenderness (22.7%) and spinal stiffness (18.2%). Blood cultures were positive in 13 cases (59.1%) represented by *Staphylococcus aureus* (31.8%). Elevated C-reactive protein levels (81.8%) and accelerated erythrocyte sedimentation rate (63.6%) were noted. Imaging features showed vertebral body osteolysis (81.8%), inflammation of adjacent soft tissue (81.8%), spinal cord compression (40.9%) and psoas abscess (13.6%). Along with medical treatment, immobilisation (72.7%), abscess drainage (13.6%) and surgery (9.1%) were indicated. The disease evolution was favourable in 20 cases (90.9%). Two patients were dead (9.1%). Sequelae were noted in 9 cases (40.9%) represented by back pain (31.8%) and spinal deformity (9.1%).

**Conclusion:**

Spondylodiscitis complicated with epidural involvement might lead to complications and sequelae if not promptly diagnosed and treated.

**Disclosures:**

**All Authors**: No reported disclosures

